# A blinded *in vitro* analysis of the intrinsic immunogenicity of hepatotoxic drugs: implications for preclinical risk assessment

**DOI:** 10.1093/toxsci/kfad101

**Published:** 2023-10-03

**Authors:** Monday O Ogese, Adam Lister, Liam Farrell, Joshua Gardner, Laila Kafu, Serat-E Ali, Andrew Gibson, Aimee Hillegas, Xiaoli Meng, Munir Pirmohamed, Geoffrey S Williams, Melanie Z Sakatis, Dean J Naisbitt

**Affiliations:** Department of Pharmacology and Therapeutic, MRC Centre for Drug Safety Science, Institute of Systems, Molecular and Integrative Biology, University of Liverpool, Liverpool L693GE, UK; Development Science, UCB Biopharma, Slough, Berkshire SL1 3WE, UK; Department of Pharmacology and Therapeutic, MRC Centre for Drug Safety Science, Institute of Systems, Molecular and Integrative Biology, University of Liverpool, Liverpool L693GE, UK; Department of Pharmacology and Therapeutic, MRC Centre for Drug Safety Science, Institute of Systems, Molecular and Integrative Biology, University of Liverpool, Liverpool L693GE, UK; Department of Pharmacology and Therapeutic, MRC Centre for Drug Safety Science, Institute of Systems, Molecular and Integrative Biology, University of Liverpool, Liverpool L693GE, UK; Department of Pharmacology and Therapeutic, MRC Centre for Drug Safety Science, Institute of Systems, Molecular and Integrative Biology, University of Liverpool, Liverpool L693GE, UK; Department of Pharmacology and Therapeutic, MRC Centre for Drug Safety Science, Institute of Systems, Molecular and Integrative Biology, University of Liverpool, Liverpool L693GE, UK; Institute for Immunology and Infectious Diseases, Murdoch University, Murdoch, Western Australia, Australia; Immunological Toxicology, In Vitro/In Vivo Translation, GSK, Collegeville, Pennsylvania, USA; Department of Pharmacology and Therapeutic, MRC Centre for Drug Safety Science, Institute of Systems, Molecular and Integrative Biology, University of Liverpool, Liverpool L693GE, UK; Department of Pharmacology and Therapeutic, MRC Centre for Drug Safety Science, Institute of Systems, Molecular and Integrative Biology, University of Liverpool, Liverpool L693GE, UK; Immunological Toxicology, In Vitro/In Vivo Translation, GSK, David Jack Centre for R&D, Ware, Hertfordshire SG12 0DP, UK; Global Investigative Safety, In Vitro/In Vivo Translation, GSK, David Jack Centre for R&D, Ware, Hertfordshire SG12 0DP, UK; Department of Pharmacology and Therapeutic, MRC Centre for Drug Safety Science, Institute of Systems, Molecular and Integrative Biology, University of Liverpool, Liverpool L693GE, UK

**Keywords:** adverse drug reaction, liver, T-lymphocytes, drug safety assessment, immunogenicity

## Abstract

*In vitro* preclinical drug-induced liver injury (DILI) risk assessment relies largely on the use of hepatocytes to measure drug-specific changes in cell function or viability. Unfortunately, this does not provide indications toward the immunogenicity of drugs and/or the likelihood of idiosyncratic reactions in the clinic. This is because the molecular initiating event in immune DILI is an interaction of the drug-derived antigen with MHC proteins and the T-cell receptor. This study utilized immune cells from drug-naïve donors, recently established immune cell coculture systems and blinded compounds with and without DILI liabilities to determine whether these new methods offer an improvement over established assessment methods for the prediction of immune-mediated DILI. Ten blinded test compounds (6 with known DILI liabilities; 4 with lower DILI liabilities) and 5 training compounds, with known T-cell-mediated immune reactions in patients, were investigated. Naïve T-cells were activated with 4/5 of the training compounds (nitroso sulfamethoxazole, vancomycin, Bandrowski’s base, and carbamazepine) and clones derived from the priming assays were activated with drug in a dose-dependent manner. The test compounds with DILI liabilities did not stimulate T-cell proliferative responses during dendritic cell-T-cell coculture; however, CD4+ clones displaying reactivity were detected toward 2 compounds (ciprofloxacin and erythromycin) with known liabilities. Drug-responsive T-cells were not detected with the compounds with lower DILI liabilities. This study provides compelling evidence that assessment of intrinsic drug immunogenicity, although complex, can provide valuable information regarding immune liabilities of some compounds prior to clinical studies or when immune reactions are observed in patients.

Drug-induced liver injury (DILI) is one of the main reasons for drug attrition and the withdrawal of already licensed drugs (Kaplowitz, 2005; Kullak-Ublick *et al.*, 2017). Many cases of DILI are delayed, idiosyncratic, immune-mediated, and therefore only affect a small percentage of individuals exposed to drugs (Chalassani *et al.*, 2014). Recent genome-wide association studies have linked expression of HLA proteins with susceptibility towards different forms of DILI. These include HLA-B*57:01 (flucloxacillin) (Daly *et al.*, 2009), HLA-A*33:03 (ticlopidine) (Hirata *et al.*, 2008), HLA-DRB1*07:01 and HLA-DQA1*02:01 (ximelagatran, lapatinib) (Kindmark *et al.*, 2008; Tangamornsuksan *et al.*, 2020), DRB1*15:01 (lumiracoxib, co-amoxiclav) (Petros *et al.*, 2017), and HLA-B*35:01/02 (green tea/minocycline) (Hoofnagle *et al.*, 2021; Urban *et al.*, 2017). Because HLA proteins are expressed by antigen presenting cells and present antigenic determinants to T-cells, the genetic associations indicate that the culprit drug is preferentially displayed by the specific risk HLA allele(s). Previously, we have shown that naïve T-cells isolated from healthy donors expressing risk alleles are activated when cultured with specific compounds in the presence of autologous dendritic cells (eg, vancomycin, HLA-A*32:01; flucloxacillin, HLA-B*57:01) (Monshi *et al.*, 2013; Ogese *et al.*, 2021); however, T-cells from donors expressing other HLA-alleles are also activated, suggesting that drugs or derived drug-proteins adducts interact with multiple HLA proteins with different degrees of specificity.

*In vitro* hepatocyte cytotoxicity assays are used alongside reactive metabolite screening and protein reactivity assays during early drug discovery to identify compounds that pose a direct DILI risk. However, the utility of these assays in predicting immune-mediated DILI can be limited. Multiple studies have identified drug-induced hepatocyte stress and the release of damage-associated molecular patterns as a potential early marker of immune-mediated hepatocyte damage (Kenna and Uetrecht, 2018). Furthermore, assays have been established to explore drug-mediated hepatocyte stress signaling to macrophages and Kupffer cells (Kato and Uetrecht, 2017; Nudischer *et al.*, 2020; Uetrecht, 2019). With additional development, these assays could be added to the battery of assays available to preclinical discovery scientists to assess DILI risk of chemicals; however, they all fail to address the pertinent question: what is the likelihood that a compound will activate effector T-cells when administered widely to humans?

Thus, the objective of this project was to evaluate the predictive value of human T-cell assays using training compounds and a set of blinded test compounds (Figure 1). The compounds were selected based on classifications of clinical hepatotoxicity status assigned by the FDA as most-, less-, or no-DILI concern and their performance in a number of hepatotoxicity-related assays in routine use by GSK (reactive metabolite formation [Sakatis *et al.*, 2012]; BSEP transporter inhibition; and *in vitro* cytotoxicity assessment [Schofield *et al.*, 2021]), in order to evaluate the potential utility, predictivity, and added value of these human T-cell assays. This resulted in 4 categories of compounds that were explored as detailed in Table 1. Compounds were coded and provided to researchers/authors in a blinded format; they were initially assessed in a peripheral blood mononuclear cell (PBMC) toxicity assay to obtain maximal individualized concentrations for immunogenicity testing. They were then used in a monocyte-derived dendritic cell-T-cell coculture to explore drug-specific naïve T-cell priming. Up to 216 individual T-cell clones were then expanded from each coculture and tested for drug specificity. A compound was characterized as potentially immunogenic if positive responses were detected in the T-cell priming experiments and/or drug-responsive T-cell clones were generated. Compounds were only unblinded once work was complete, and are presented here in an unblinded narrative.

**Figure 1.**
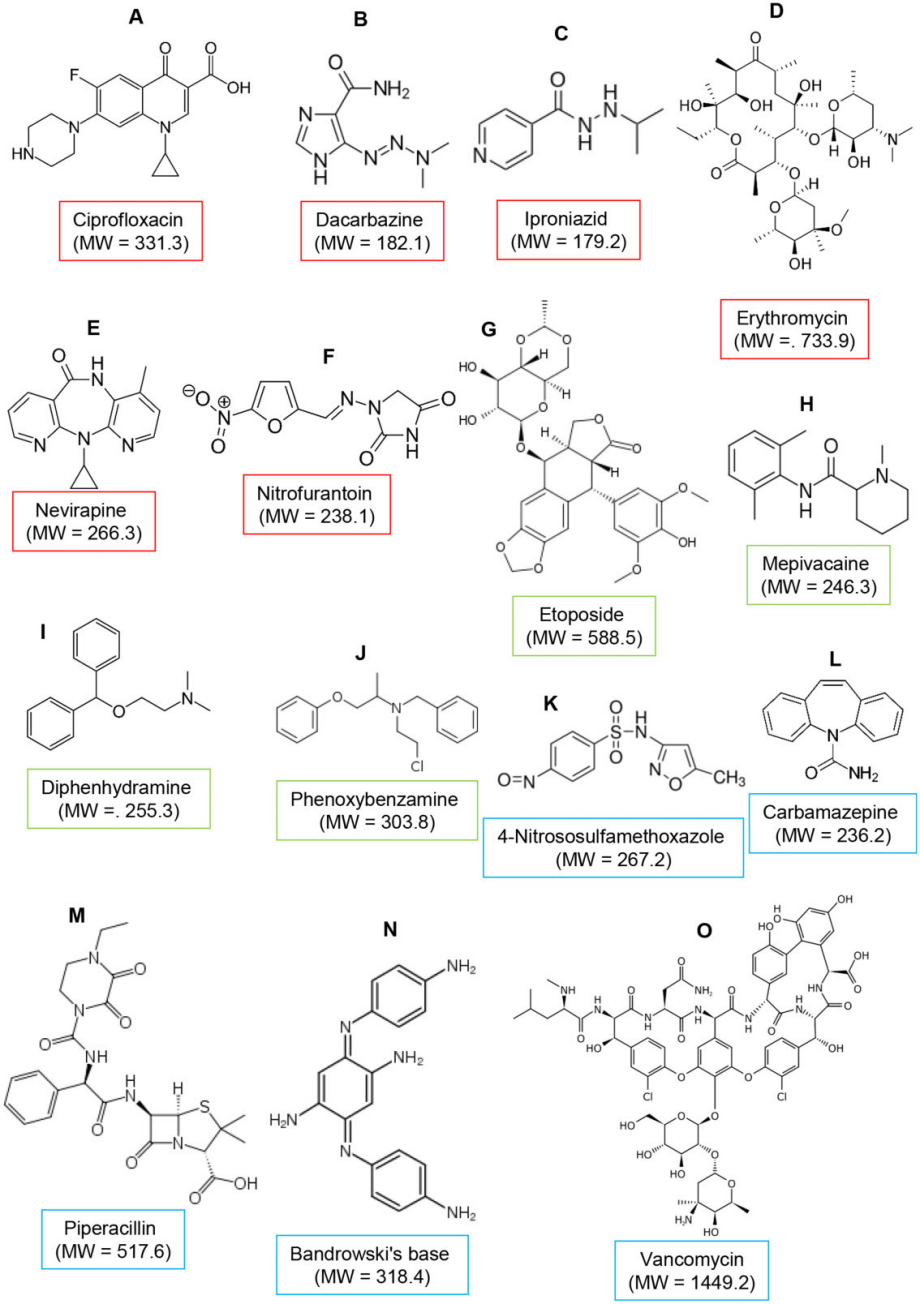
Compounds utilized for immunogenicity studies. Training compounds in blue, test compounds with DILI risk in red and test compounds with low DILI risk in green.

**Table 1.** Details of test compounds

Category and Rationale	Drug Identity	**Maximum Clinical Dose** ^a^ **(mg/day)**	**FDA Clinical Hepatotoxicity Concern** ^b^	**Routine Assays that are Positive (GSK)** ^c^	**Relevant Annotations from National Institute of Diabetes and Digestive and Kidney Diseases (2012),** https://www.ncbi.nlm.nih.gov/books/NBK547852/ **(Unless Stated Otherwise)**
(i) Most-DILI concern missed by GSK hepatotoxicity screening assays—to assess potential added value of T-cell assays	Ciprofloxacin^d^	1500	Most	None	Likely cause of clinically apparent liver injury. Allergic manifestation present—fever, rash, eosinophilia. Mechanism suspected to be hypersensitivity
	Iproniazid	250	Most	None	Not in LiverTox and no drug label available (withdrawn). Mechanism of injury proposed to be via reactive metabolite mechanisms (Nelson *et al.*, 1976; Spearman *et al.*, 1984) not flagged in typical screening assays
	Nevirapine	400	Most	None	Well-known cause of clinically apparent liver injury. Immunoallergic hepatitis associated with specific HLA types
(ii) No-DILI concern, negative in all GSK hepatotoxicity screening assays—to assess potential for false positives in T-cell assays	Diphenhydramine	300	No	None	Unlikely to be a cause of clinically apparent liver injury
	Mepivacaine	400	No	None	Unlikely to be a cause of clinically apparent liver injury
(iii) Most-DILI concern with positives in reactive metabolite screening assays—to assess value of T-cell assays in this scenario	Dacarbazine	608	Most	Reactive metabolite; 3D hepatocyte spheroid	Highly likely cause of clinically apparent liver injury. May be immunologically mediated as usually occurs with second or third cycle and accompanied by eosinophilia
	Erythromycin	4000	Most	Reactive metabolite	Well-known cause of clinically apparent liver injury. Idiosyncratic. Mechanism assumed to be hypersensitivity due to allergic manifestations (rash, fever, eosinophilia) and that can occur sooner, or for first time, on rechallenge
	Nitrofurantoin	400	Most	Reactive metabolite	Well-known cause of clinically apparent liver injury. Oxidative free radical formation. Some cases show autoimmune etiology with linkage to HLA-DR6 and DR2
(iv) No- or Less-DILI concern with positives in reactive metabolite screening assays—to assess potential for false positives and utility in this scenario	Etoposide	300	Less	Reactive metabolite	Probable cause of clinically apparent liver injury. Absence of immunoallergic features (rash, fever, eosinophilia) and of autoantibodies noted. Uncommon to have features of hypersensitivity
	Phenoxybenzamine	120	No	Reactive metabolite	Not in LiverTox. No mention of hepatotoxicity or immunoallergic manifestations on the label

a The maximum clinical daily dose was obtained from the drug label when available. For withdrawn drugs, doses were obtained from the National Institute of Diabetes and Digestive and Kidney Diseases database (2012), the FDA LTKB benchmark dataset (Chen *et al.*, 2011b) or published journal articles.

b Hepatotoxicity classifications reported by the FDA as part of DILIrank datasets (for drugs with confirmed causal evidence linking the drug to liver injury) of most-DILI-concern (withdrawn or discontinued, have a boxed warning, or have severe DILI content in the warning and precaution section of the drug label), less-DILI-concern (mild DILI content in the warnings and precautions section or DILI events highlighted in the Adverse Reactions section of the drug label) and no-DILI-concern (drug label do not contain any DILI event).

c Routine GSK screening assays: Reactive metabolite formation assays (GSH adduct formation, CYP3A4 metabolism dependent inhibition); bile salt export pump (BSEP) inhibition; *in vitro* cytotoxicity assays (3D-hepatocyte spheroid assay or 2D HepG2 cell health assay). “Positive” is defined as above the threshold of concern for each assay. All compounds have been tested in all assay types, with only those that were positive being listed, and all other assays not listed being negative (below the threshold of concern for the assay).

d Table describes features of test compounds. Adverse reactions associated with training compounds have been extensively described and thus are excluded from the table.

## Materials and methods

###  

####  

##### Establishment of a PBMC bio-bank

This study was approved by the local NHS Research Ethics Committee and all participants gave written informed consent before the study commenced. Recruitment of the volunteers was undertaken by research nurses at the University of Liverpool and each agreed to donate 100 ml of blood. The ethical approval contains an option to recall individuals to donate additional fresh blood, if required for further studies. PBMC were isolated using density centrifugation and cryopreserved at −150°C. PBMCs from 3 donors were selected at random for testing using the dendritic cell, T-cell coculture, with the same 3 donors used to assess 2–3 compounds depending on the availability of cells. PBMCs from the same 3 donors were utilized for the generation of T-cell clones against all of the compounds; HLA genotype characterization performed by Histogenetics Laboratory (New York) is presented in Table 2.

**Table 2.** HLA genotype of drug-naïve healthy donors utilized for the generation of drug-specific T-cell clones

Subject ID	HLA Class I						HLA Class II					
	HLA-A		HLA-B		HLA-C		HLA-DRB1		HLA-DQB1		HLA-DQA1	
Donor 1	01:02	26:01	40:01	49:01	03:04	07:01	04:04	07:01	02:01	03:02	02:01	03:01
Donor 2	02:01	03:01	44:03	44:02	05:01	16:01	07:01	15:01	02:01	06:02	01:02	02:01
Donor 3	11:01	30:01	13:02	35:01	04:01	06:02	15:01	16:01	05:02	06:03	01:02	01:02

##### Cell culture medium, reagents, and training and test compounds

All PBMC and purified immune cells were cultured in RPMI 1640 medium, containing 10% human serum (blood type AB), 100 mM l-glutamine, 25 mM HEPES, 100 µg/ml penicillin and 100 U/ml streptomycin, and 25 µg/ml transferrin (Sigma-Aldrich; Dorset, UK). Magnetic beads for cell isolations were purchased from Miltenyi Biotec Ltd. (Bisley, UK). CD4-APC and CD8-PE antibodies used for flow cytometry were purchased from BD Biosciences (Oxford, UK). Training and test compounds were sourced from standard commercial suppliers.

##### Inhibition of mitogen-induced PBMC proliferation using training and test compounds

Test compounds were provided by GSK to Liverpool as blinded dry powders with a given volume of dimethyl sulfoxide to prepare stock concentrations of 50 mM. Stock concentrations were aliquoted and stored at −80°C until required for experiments. A fresh aliquot was utilized for every experiment. PBMC from 3 healthy donors were used to determine the maximum tolerated concentration of each test compound. PBMC (0.15 × 10^6^) were cultured with graded compound concentrations in U-bottomed 96-well plates for 48 h at 37°C, 5% CO_2_. Phytohemagglutinin (PHA-P; 10 µg/ml) was added to cells for a further 24 h followed by assessment of proliferation through the addition of [^3^H]-thymidine (0.5 µCi/well) for the last 16 h. Cells were harvested using TomTec Harvester 96 (Receptor Technologies) onto filter mats, sealed with scintillation wax and PBMC proliferation determined using a MicroBeta TriLux 1450 LSC β-counter (PerkinElmer). The IC_50_ value was then calculated using 4-parameter logistic curve-fitting model (GraphPad prism 7.0).

##### Priming of naïve human T-cells to training and test compounds

To investigate the intrinsic immunogenicity of the training and test compounds, monocytes and naïve T-cells were isolated from healthy donor PBMC using an established protocol (Ogese *et al.*, 2020). CD14+ monocytes were positively selected using CD14 antibody-conjugated microbeads. This was followed by the depletion of CD25+ cells from the CD14- population and the negative selection of CD45RA+ naïve T-cells. Naïve T-cells with a purity of greater than 97% were stored at −150°C freezer while CD14+ monocytes were used to generate dendritic cells. Dendritic cells were generated by culturing monocytes with a cocktail of GM-CSF (800 U/ml) and IL-4 (800 U/ml) in culture media for 6 days. Both cytokines were purchased from Peprotech (New Jersey). A maturation cocktail of TNF-α (25 ng/ml) and LPS (1 µg/ml) was added for 16 h before establishing the T-cell priming culture. Dendritic cells (8 × 10^3^ per well) were cultured with naïve T-cells (1 × 10^5^ per well) and the test compounds in a 96-well U-bottom tissue culture plate for 14 days at 37°C, 5% CO_2_. Cells were then washed extensively to remove unbound drug and cultures were restimulated with test compound or medium control (48 wells) for 48 h. Subsequent experiments were performed with training compounds to investigate the dose-dependent stimulation of naïve T-cells. In these experiments, T-cells were cultured with 3 concentrations of the test compound and medium (negative control) during the restimulation step. T-cell proliferative responses were determined by addition of [^3^H]-thymidine (0.5 µCi/well) for the final 16 h of the culture period. Plates were harvested and counted as described above. The degree of naïve T-cell priming is displayed as counts per minute in individual wells. Student’s *t* test was performed to determine statistical significance of T-cell proliferation when comparing medium- and drug-treated cultures (*p ≤ .05; **p ≤ .005; ***p < .001).

##### Generation of Epstein-Barr virus-transformed B-cell lines and drug-specific T-cell clones against training and test compounds

Epstein-Barr virus (EBV)-transformed autologous B-cell lines were generated for use as antigen presenting cells (Zhao *et al.*, 2021). Briefly, PBMC were cultured with supernatant from the EBV-producing cell line B9-58 for 16 h. The cells were then centrifuged and maintained in B-cell culture medium consisting of RPMI supplemented with bovine serum albumin (10%) penicillin (100 µg/ml), streptomycin (100 U/ml), HEPES buffer (25 mM), and l-glutamine (2 mM). Cyclosporin A was included in the medium for the first 3 weeks to prevent T-cell outgrowth.

**Figure 2.**
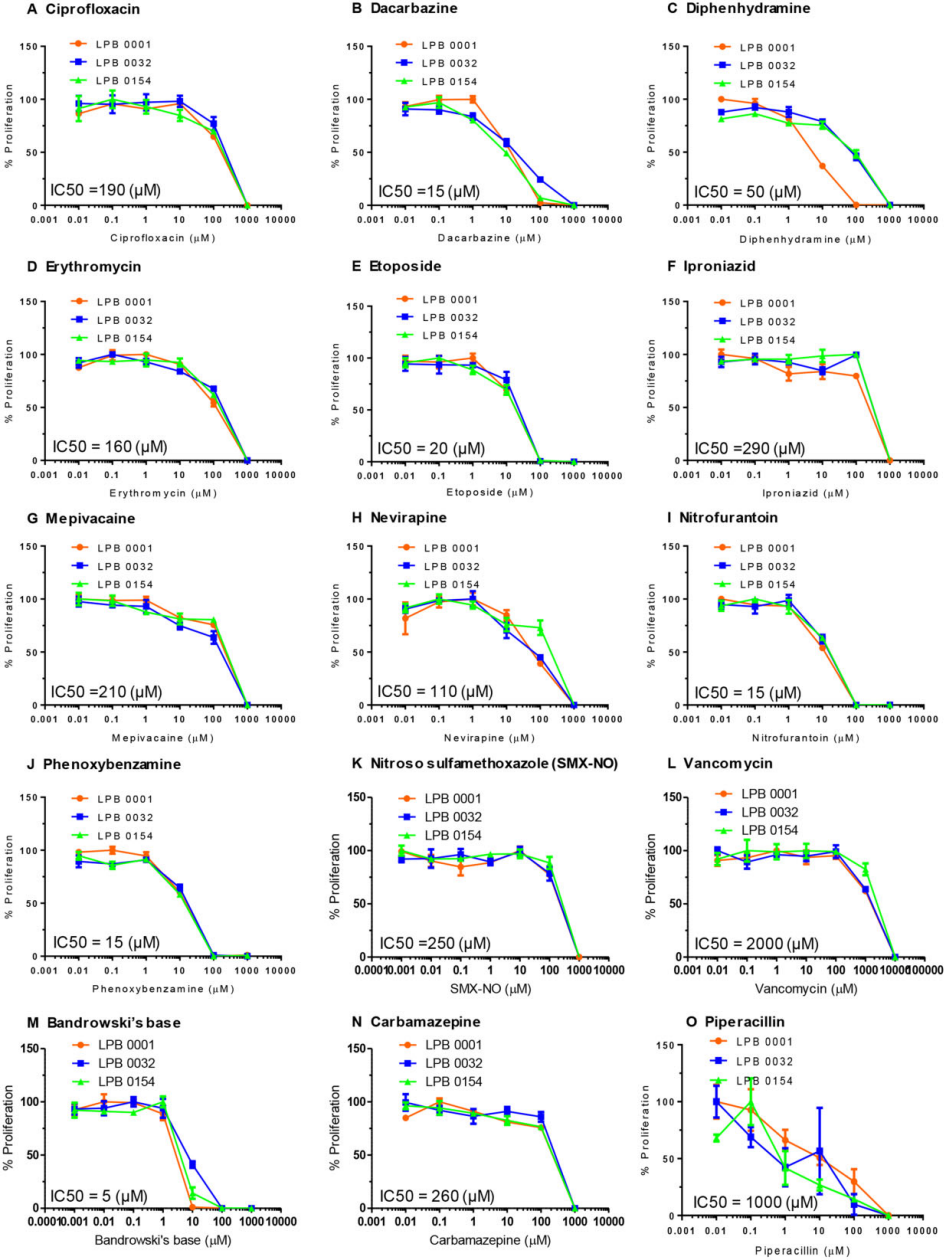
Toxicity profiles of training and test compounds. PBMC (0.15 × 10^6^) from 3 healthy donors (LPB 001, LPB 0032, and LPB 0154) were cultured with graded compound concentrations in U-bottomed 96-well plates for 48 h at 37°C, 5% CO_2_. PHA-P (10 µg/ml) was added to cells for a further 24 h followed by assessment of proliferation through the addition of [^3^H]-thymidine (0.5 µCi/well) for the last 16 h. PBMC proliferation determined using a MicroBeta TriLux 1450 LSC β-counter. 4-parameter logistic functions were fit to the data and IC_50_ values were then reported (GraphPad prism 7.0). A–J, test compounds; K–O, training compounds. LPB (Liverpool Pharmacology Biobank) represents the unique identifier for PBMC isolated from each consented healthy blood donor for drug safety research. Percentage proliferation for each compound concentration = Proliferation following drug exposure divided by proliferation after cell culture media exposure multiplied by 100.

To establish T-cell lines, PBMC from 3 healthy HLA-typed (Table 2) donors were cultured with individual test compounds (at concentrations not associated with inhibition of mitogen-driven PBMC proliferation; see Figure 2 for individual concentrations) for 14 days. Medium was supplemented with IL-2 (50 U/ml; Peprotech, New Jersey) to maintain drug-specific T-cell expansion. T-cell clones were generated from the T-cell lines by serial dilution and repetitive mitogen stimulation (Schnyder *et al.*, 2000). T-cells (0.3–3 cells/well) were cultured with irradiated allogenic PBMC (5 × 10^4^/well) and PHA-P (1 µg/ml) in IL-2 containing medium. The medium was supplemented with fresh IL-2 on days 6, 9 and every 2 days thereafter. Growing cultures were expanded further with a second round of mitogen stimulation and tested for drug-specific T-cell proliferation on day 28. Cloned T-cells (5 × 10^4^/well) were cultured with irradiated autologous EBV-transformed B-cells (1 × 10^4^/well) and drug in duplicate wells for 48 h. Wells containing medium served as a negative control. Proliferation was measured by the addition of [^3^H]-thymidine followed by scintillation counting. T-cell clones with SI ≥ 2 were considered drug-responsive and expanded using irradiated allogeneic PBMC for dose-response studies and assessment of CD4/8 phenotyping.

##### Assessment of dose-dependent, compound-specific proliferation of T-cell clones

Dose-dependent drug-specific activation of T-cell clones was performed by incubating T-cell clones (5 × 10^4^/well) with irradiated EBV-transformed B-cells (1 × 10^4^/well) and titrated concentrations of test compounds for 48 h in triplicate wells at 37°C, 5% CO_2_. Proliferation was measured by the addition of [^3^H]-thymidine as described above.

##### Phenotypic analysis of T-cell clones

Compound-responsive T-cell clones were incubated with 3 and 1 µl of CD4 (clone RPA T4, APC) and CD8 (clone HIT8a, PE) fluorochrome-conjugated antibodies, respectively on ice for 20 min. T-cell clones were washed with FACS buffer and CD phenotype determined using flow cytometry. Cells (10 000) were acquired using a FACSCanto II (BD Biosciences) and data analyzed by FACSDiva software.

## Results

###  

#### Training and test compounds inhibit mitogen-driven PBMC proliferation over a range of concentrations

Assessment of mitogen-driven proliferation with the training and test compounds was performed to determine the optimum study concentration for the T-cell stimulation studies. PBMC from 3 healthy donors were cultured with titrated concentrations of each compound and IC_50_ values were calculated. The concentration of compound associated with 50% inhibition of mitogen-driven PBMC proliferation ranged from 5 to 2000 µM (Figs. 2A–O). Vancomycin and Bandrowski’s base showed the highest and lowest IC_50_ values, 2000 µM and 5 µM, respectively. Dacarbazine, etoposide, nitrofurantoin, and phenoxybenzamine displayed IC50 concentrations ≤20 µM. In addition, ciprofloxacin, diphenhydramine, erythromycin, iproniazid, mepivacaine, nevirapine, nitroso sulfamethoxazole, and carbamazepine had IC_50_ values ≥ 50 µM.

#### Determination of naïve T-cell priming with training and test compounds

**Figure 3.**
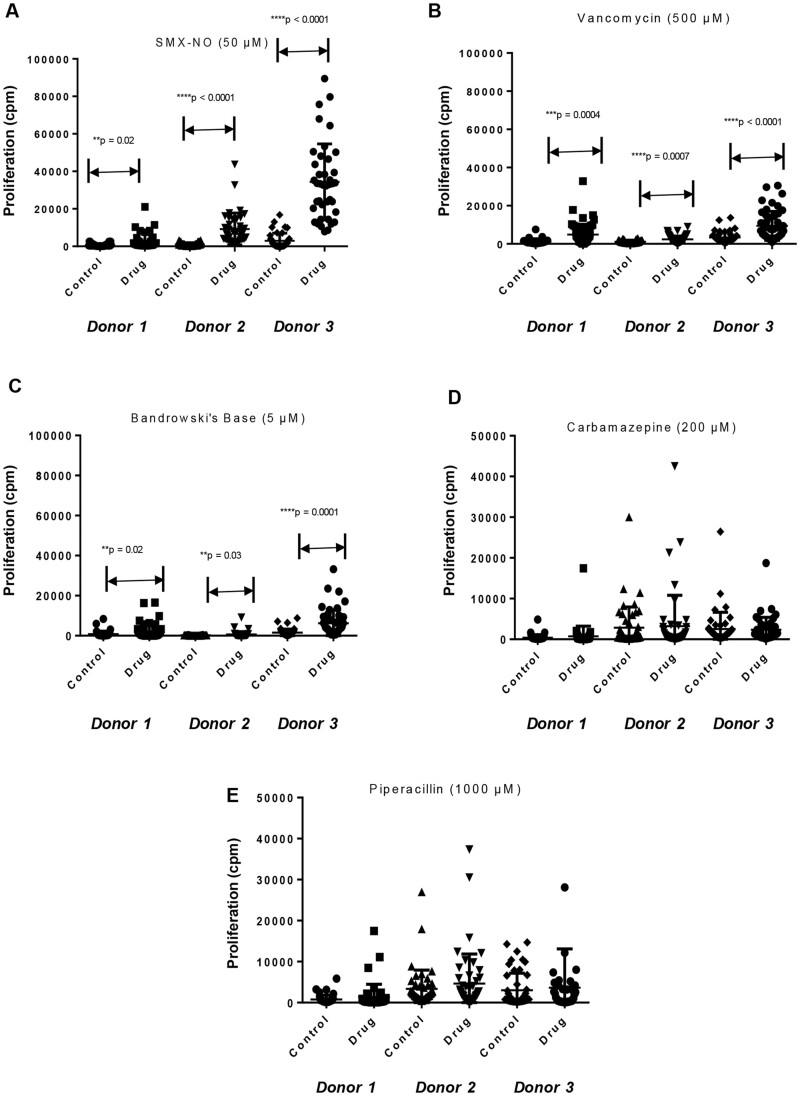
Naïve T-cell priming with training compounds. CD14+ monocytes were utilized to generate dendritic cells with a cocktail of GM-CSF (800 U/ml) and IL-4 (800 U/ml) in culture media for 6 days. A maturation cocktail of TNF-α (25 ng/ml) and LPS (1 µg/ml) was added to dendritic cells for 16 h before the coculture of naïve T-cells, dendritic cells, and individual test drug (≤IC50). The test concentrations used for naive T-cell priming are displayed as chart title on the top of each graph. A, Nitroso sulfamethoxazole. B, Vancomycin. C, Bandrowski’s base. D, Carbamazepine. E, Piperacillin. The degree of naïve T-cell priming was determined by comparing the counts per minutes (cpm) of drug and media-treated cultures using a beta counter. Student’s *t* test was performed to determine statistical significance (*p ≤ .05; **p ≤ .005; ***p < .001). Each data point represents T-cells proliferation in individual well of a 96 U-bottomed plate.

**Figure 4.**
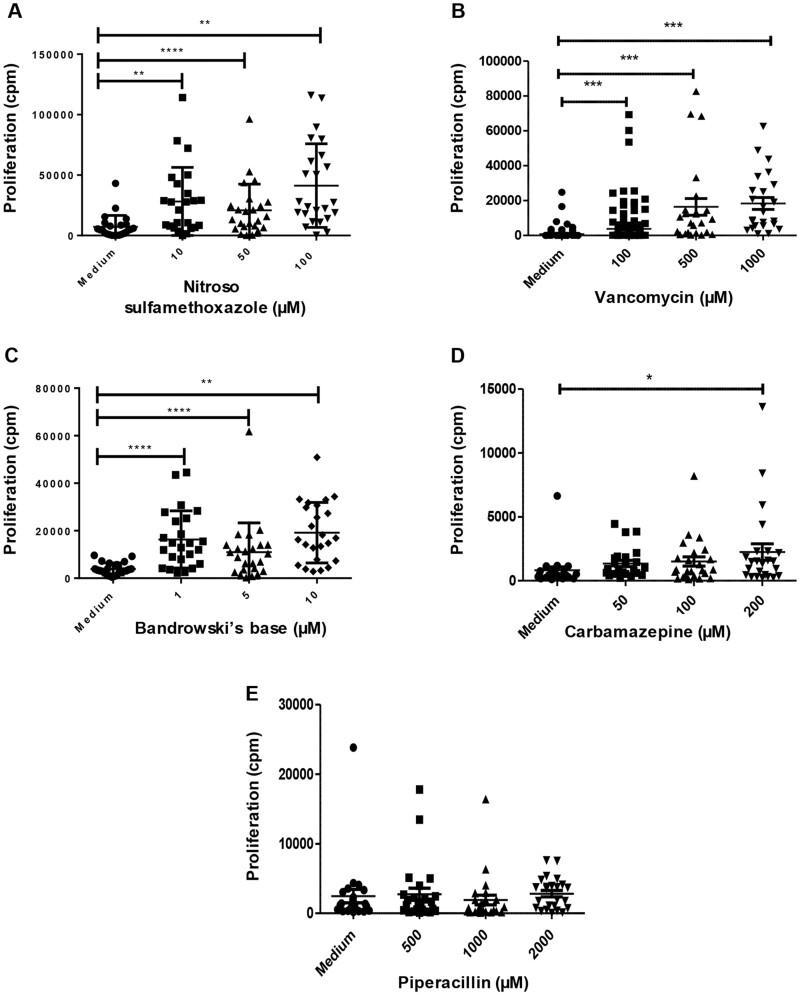
Dose-dependent stimulation of primed T-cells with training compound. Monocyte-derived dendritic cells and naïve T-cells were cultured with the optimum concentrations of (A) nitroso sulfamethoxazole (50 µM), (B) vancomycin (500 µM), (C) Bandrowski’s base (5 µM), (D) carbamazepine (200 µM), or (E) piperacillin (1000 µM) for 2 weeks as described in Figure 3 legend. Primed cells were washed extensively and then restimulated with 3 drug concentrations (low, medium, and high) for 48 h followed by determination of T-cell activation by [^3^H]-thymidine incorporation. Each data point represents T-cells proliferation in individual well of a 96 U-bottomed plate.

Naïve T-cells and dendritic cells were cultured with the training compounds for 2 weeks to measure T-cell priming responses. Proliferation of primed T-cells was observed for 3 of the training compounds (nitroso sulfamethoxazole, Bandrowski’s base, and vancomycin) when the strength of proliferation for 48 wells restimulated with compound was compared with wells without compound re-stimulation (Figs. 3A–C). In contrast, specific proliferation of primed T-cells was not observed for carbamazepine or piperacillin-treated (Figs. 3D and 3E). Experiments were repeated, with the primed T-cells being restimulated with titrated concentrations of the training compounds, and similar results were observed (Figure 4). Naive T-cells primed against nitroso sulfamethoazole, Bandrowski’s base, or vancomycin were stimulated to proliferate in the presence of all 3 concentrations of the training compounds. Carbamazepine-primed T-cells displayed a weakly significant proliferative response when restimulated at the highest concentration of 200 µM. Proliferation of piperacillin primed T-cells was not detected.

**Figure 5.**
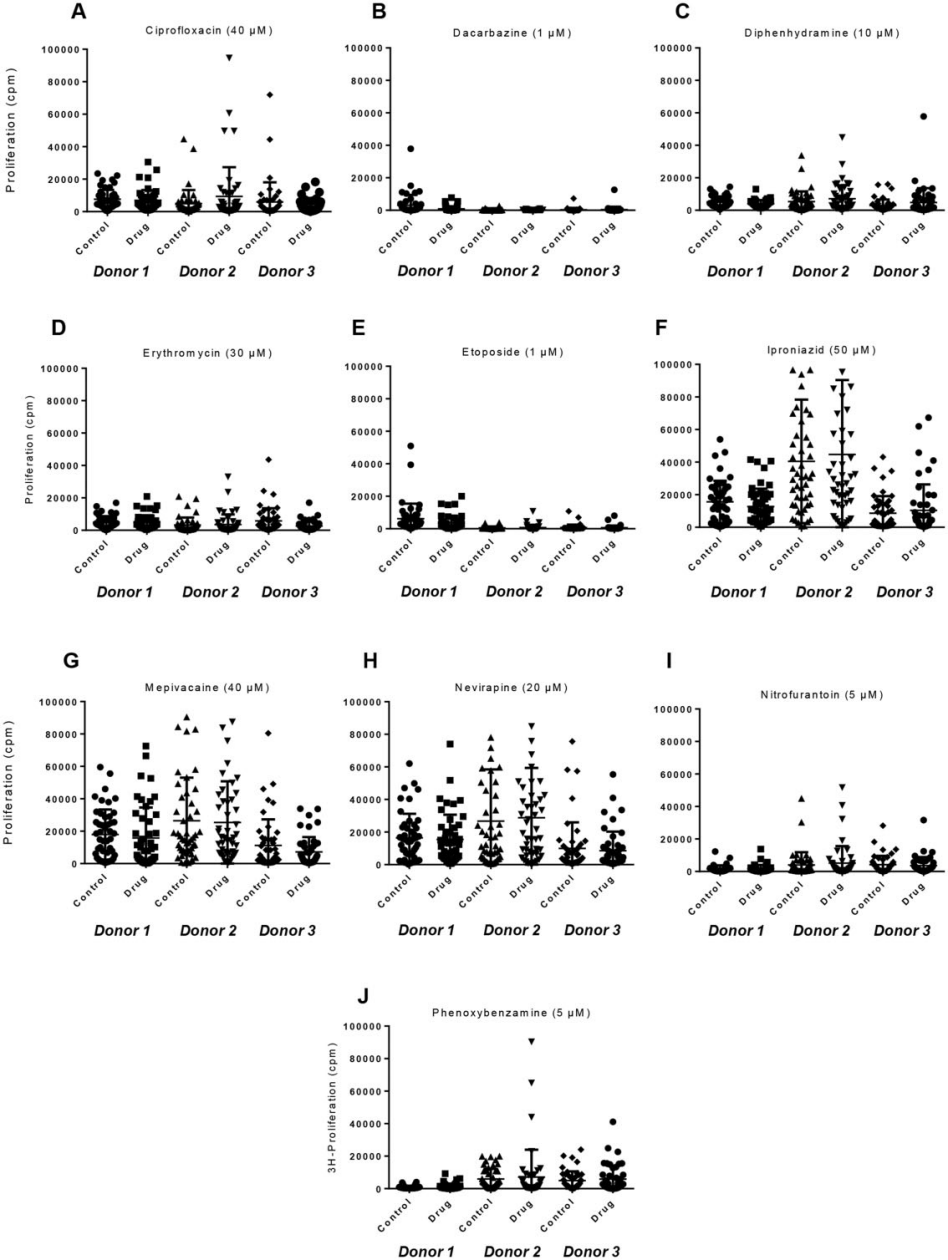
Naïve T-cell priming with test compounds. Monocyte-derived dendritic cells and naïve T-cells were cultured with the maximum nontoxic concentrations of test compounds using the method described in Figure 3 legend. A, Ciprofloxacin. B, Dacarbazine. C, Diphenhydramine. D, Erythromycin. E, Etoposide. F, Iproniazid. G, Mepivacaine. H, Nevirapine. I, Nitrofurantoin. J, Phenoxybenzamine. Primed cells were washed extensively and then restimulated medium or drug for 48 h followed by determination of T-cell activation by [^3^H]-thymidine incorporation. Each data point represents T-cells proliferation in individual well of a 96 U-bottomed plate.

None of the test compounds showed drug-specific T-cell priming as evidenced through increased proliferative responses when comparing drug- and medium-restimulated cells (Figs. 5A–J). Naïve T-cells primed against iproniazid, mepivacaine, or nevirapine for 2 weeks displayed a high level of proliferation in medium restimulated wells, which was not seen with other compounds; however, increases in proliferation with drug restimulation was not observed.

#### Characterization of drug-specific T-cell clones

**Figure 6.**
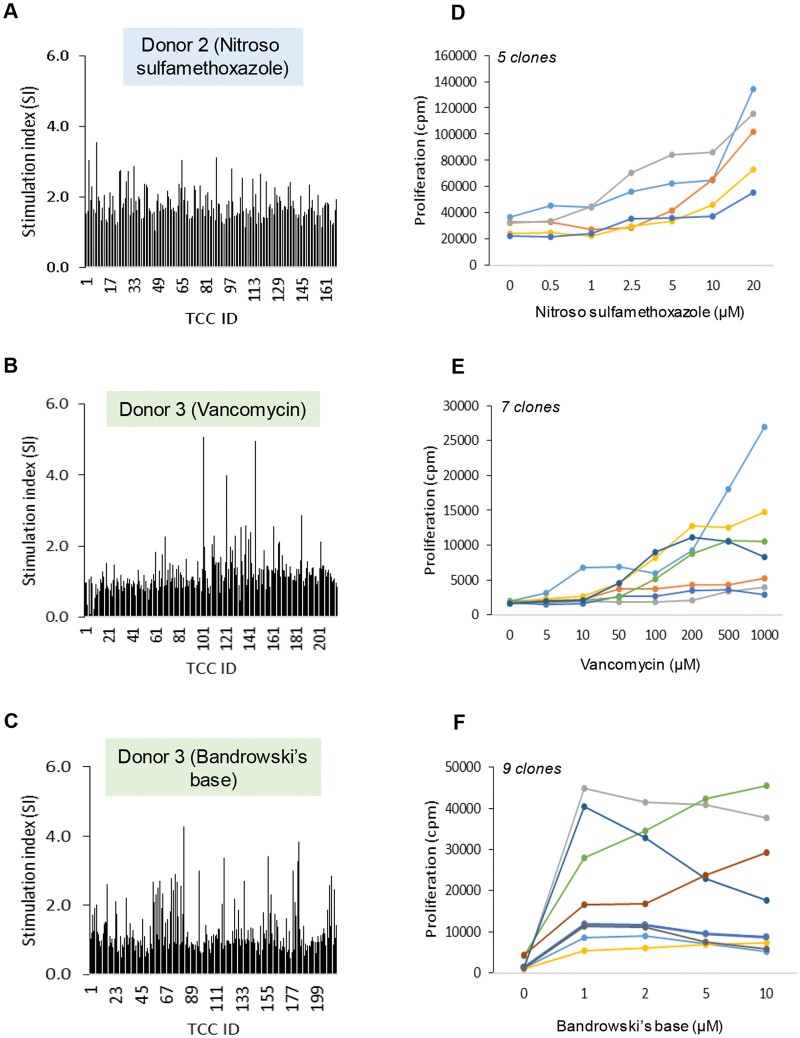
Generation of training compound (nitroso sulfamethoxazole, vancomycin, and Bandrowski’s base)-specific T-cell clones. PBMC from 3 healthy donors were cultured with training compounds and supplemented with IL-2 (50 U/ml) for 14 days. T-cell clones were generated from the T-cell lines by serial dilution and repetitive mitogen stimulation. T-cells (0.3–3 cells/well) were stimulated with irradiated allogenic PBMC (5 × 10^4^/well) and phytohemagglutinin (1 µg/ml). Well growing cultures were subjected to a second round of expansion then tested for drug-specific T-cell proliferation. Proliferation was measured by the addition of ^3^H-thymidine followed by scintillation counting. A–C, Initial specificity test for nitroso sulfamethoxazole, vancomycin, and Bandrowski’s base clones. T-cell clones showing stimulation index, SI ≥ 2 were considered drug-responsive and expanded and used in dose-response studies. SI = Drug-induced T-cell proliferation divided by T-cell proliferation in cell culture media. D–F, Dose-dependent activation of nitroso sulfamethoxazole (*n* = 5), vancomycin (*n* = 7), and Bandrowski’s base (*n* = 8)-responsive T-cell clones. T-cell activation was determined using [^3^H]-thymidine incorporation.

A maximum of 216 T-cell clones per donor (3 donors per drug) were expanded and tested for drug specificity. From the drug specificity testing, 10.0%, 9.7%, and 13% were responsive to nitroso sulfamethoxazole, vancomycin, and Bandrowski’s base, respectively. Figure 6 shows initial testing of all clones from 1 representative individual (medium, optimal drug concentration in duplicate) and then dose-response analysis (triplicate incubations) of a panel of rapidly expanding clones for each compound. Carbamazepine- and piperacillin-responsive T-cell clones were not detected.

**Figure 7.**
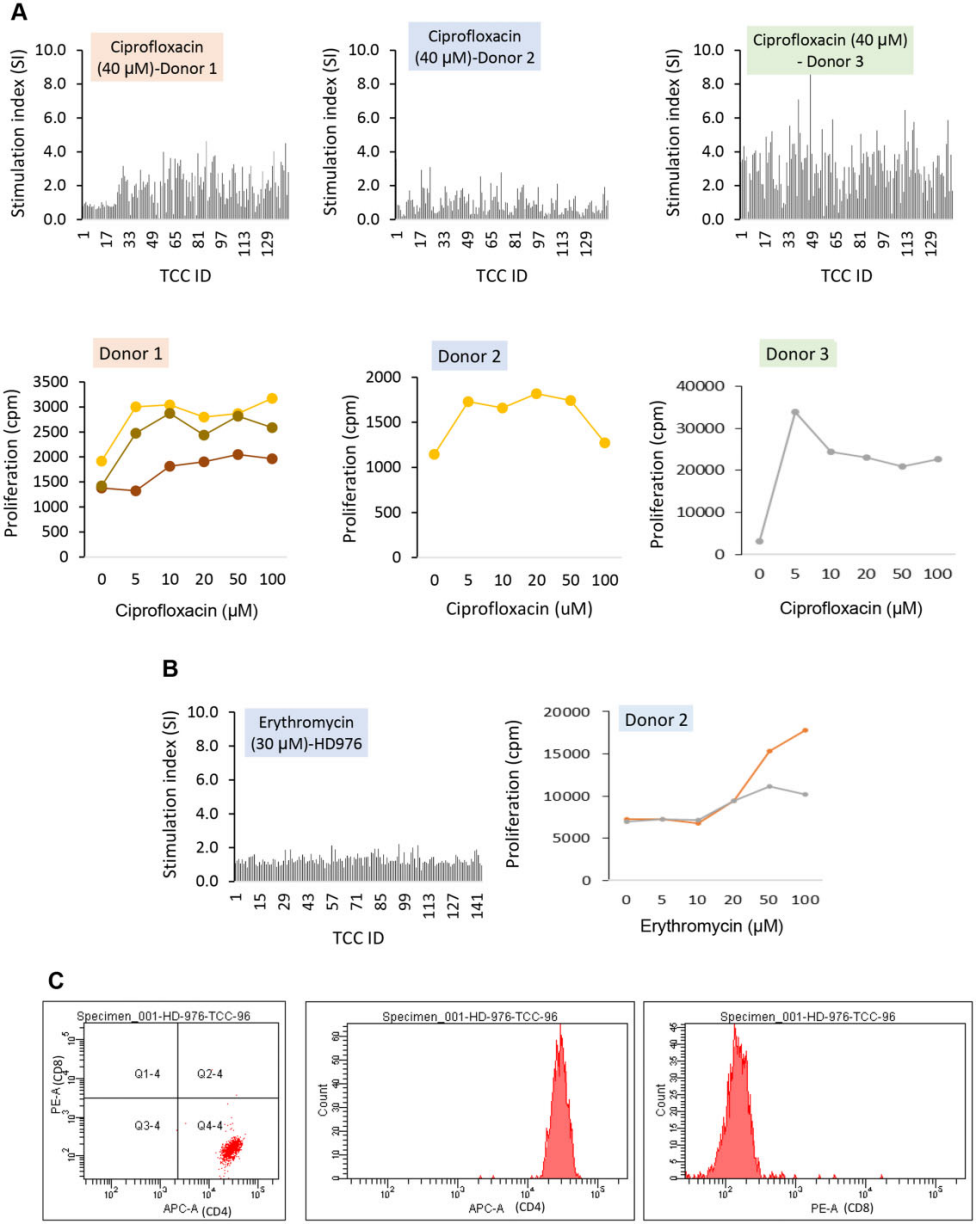
Generation of test compound (ciprofloxacin and erythromycin)-specific T-cell clones. PBMC from 3 healthy donors were cultured with test compounds and supplemented with IL-2 (50 U/ml) for 14 days. T-cell clones were generated from the T-cell lines by serial dilution and repetitive mitogen stimulation. T-cells (0.3–3 cells/well) were stimulated with irradiated allogenic PBMC (5 × 10^4^/well) and phytohemagglutinin (1 µg/ml). Well growing cultures were subjected to a second round of expansion then tested for drug-specific T-cell proliferation. Proliferation was measured by the addition of ^3^H-thymidine followed by scintillation counting. A, Initial specificity test for ciprofloxacin, using PBMC from 3 donors followed by dose-dependent activation of 5 clones. B, Initial specificity test for erythromycin, using PBMC from 1 donor followed by dose-dependent activation of 2 clones. T-cell activation was determined using [^3^H]-thymidine incorporation. C, CD phenotyping of a representative erythromycin-specific CD4+ T-cell clone.

For the test compounds, a maximum of 144 T-cell clones were generated from each donor and tested for drug specificity. Ciprofloxacin stimulated 43%, 5.5%, and 75.7% of T-cell clones from donors 1 to 3, respectively, to proliferate (Figure 7 and Supplementary Figure 1 shows initial testing responses from all 3 donors). Five well-growing CD4+ ciprofloxacin-responsive T-cell clones selected from each of the 3 donors displayed dose-dependent proliferative responses in the presence of ciprofloxacin. Only 3 erythromycin-responsive T-cell clones, representing 2% of the total clones tested, were identified from donor 2 (Figure 7). Erythromycin-responsive clones were not detected from the other donors (Supplementary Figure 1). Two of the erythromycin-responsive clones expressed CD4+ and displayed dose-dependent proliferative responses when expanded and assayed with multiple concentrations of erythromycin (Figure 7). Drug-specific T-cell clones were not identified for the other test compounds (Supplementary Figure 1); hence, none of these clones were tested further.

In summary, although the 6 test compounds with known DILI liabilities did not induce detectable T-cell proliferation in the dendritic cell-T-cell co-culture, CD4+ clones displaying reactivity were detected for 2 of these compounds (ciprofloxacin and erythromycin), whereas no drug-responsive T-cell proliferation or drug-specific T-cell clones were detected for the 4 compounds with lower DILI liabilities.

## Discussion

A small portion of the human population develop serious and potentially life-threatening T-cell-mediated adverse drug reactions. This can result in restrictions of a drug’s use and sometimes drug withdrawal. DILI is one example of an immune-mediated reaction that cannot easily be predicted preclinically from the known chemistry or pharmacology of the compound in question and thus, when reactions appear, they represent a significant challenge to clinicians, drug safety scientists, and drug regulatory agencies. No clear dose-response relationship exists in most cases of immune-mediated DILI; in fact, patients that develop an adverse event have typically received the same dose of a particular drug as patients that tolerate the drug. As the molecular initiating event in immune-mediated DILI involves drug, drug metabolite *or* drug-modified peptide (derived from processed drug hapten protein adducts) being presented on HLA molecules and subsequent recognition by TCRs, reactive metabolite screens and direct hepatotoxicity/cell stress assays do not adequately predict the likelihood that a compound will cause an adverse event. Hence, drug-specific human T-cell assays may have applicability in predicting of drug immunogenicity.

Newman *et al.* (1977) demonstrated that human lymphoid cells could be primed *in vitro* against chemical sensitizers. Seldin and Rich (1978) then developed a model to study responses of lymphocytes to hapten protein conjugates. Coculture of lymphocytes with hapten-modified PBMC resulted in T-cell proliferation in an antigen-specific manner. *In vitro* T-cell priming methods have been modified in several subsequent studies (Krasteva *et al.*, 1996; Rougier *et al.*, 1998, 2000; Rustemeyer *et al.*, 1999); specifically, different forms of dendritic cell have been used to present the antigenic material with naïve T-cells as responders. These assays discriminated strong sensitizers from irritants; however, weak sensitizers did not stimulate a T-cell response. In recent years, improved protocols have been used to generate more stimulatory dendritic cells, and regulatory T-cells have been removed from responder cells to enhance sensitivity. Furthermore, multiple readouts are now available to detect the antigen-specific T-cell response (Dietz *et al.*, 2010; Martin *et al.*, 2010; Richter *et al.*, 2013; Vocanson *et al.*, 2008). We have recently applied similar dendritic cell-T-cell cocultures to assess the immunogenicity of drugs. Structurally divergent drugs that activate T-cells via direct drug or metabolite HLA binding, and the formation of hapten protein conjugates have been shown to activate naïve CD4+ and CD8+ T-cells (Faulkner *et al.*, 2012, 2016; Gibson *et al.*, 2017a; Ogese *et al.*, 2019; Usui *et al.*, 2018). Interestingly, modulation of the immune regulatory network *in vitro* using checkpoint blockade (PD-L1 CTLA-4 mAbs) increases the likelihood that drug-responsive T-cells are detected (Gibson *et al.*, 2014, 2017b), although these mAbs also increase background proliferation and nonantigen-specific cytokine release. In recent years, we have focused on (1) simplifying the experimental design by removing the requirement for a second population of dendritic cells when restimulating primed T-cells, (2) demonstrating that drug-specific T-cell priming responses are detectable using PBMC from donors with and without HLA risk alleles, and (3) increasing the number of experimental replicates to obtain more accurate readouts, (4) generating T-cell clones to characterize drug-specific T-cell phenotype and function, and assess cross-reactivity (Alhilali *et al.*, 2019; Alzahrani *et al.*, 2017; Hammond *et al.*, 2021; Ogese *et al.*, 2020, 2021; Usui *et al.*, 2018b).

The objective of this project was to utilize an established dendritic cell-T-cell coculture, and T-cell cloning methods, to assess antigen-specific T-cell responses induced by drugs associated with human DILI. This could potentially be used to prospectively assess the potential for T-cell activation of a compound and thus the potential for DILI via this mechanism, that would otherwise not have been captured by hepatotoxicity-related screening assays typically used within the pharmaceutical industry. To that end, test compounds were selected and blinded, and used alongside training compounds associated with clinically and immunologically well-described adverse events.

To allow compounds to be added to the dendritic cell-T-cell coculture at a single concentration, initial experiments were performed to define the maximum concentration of each compound that did not inhibit mitogen-induced PBMC proliferation. From these experiments, concentrations ranging from 1 to 1000 µM were selected for the T-cell priming experiments. Three of the training compounds, nitroso sulfamethoxazole, vancomycin, and Bandrowski’s base, induced proliferation of naïve T-cells from all 3 study donors. Multiple wells from each priming experiment contained T-cells that were stimulated to proliferate in a dose-dependent manner. Nitroso sulfamethoxazole is a cysteine-reactive metabolite of the antibacterial agent sulfamethoxazole that stimulates T-cells isolated from patients with allergic drug reactions targeting skin (Castrejon *et al.*, 2010; Schnyder *et al.*, 2000), and is commonly used as a positive control for *in vitro* T-cell priming experiments (Faulkner *et al.*, 2012, 2016). Vancomycin is a glycopeptide antibiotic that activates T-cells isolated from patients with drug reaction with eosinophilia and systemic symptoms following vancomycin treatment (Nakkam *et al.*, 2021), and has been shown to activate CD4+ and CD8+ T-cells from healthy donors (irrespective of whether they express the HLA risk allele A*32:01; Konvinse *et al.*, 2019), via a direct binding interaction with HLA and T-cell receptors (Ogese *et al.*, 2021). Bandrowski’s base is a trimer of *p*-phenylenediamine, which is found widely in the environment, for example in black clothing, inks, hair dye, dyed fur/leather and photographic products. We have previously shown that Bandrowski’s base-responsive T-cells are detectable in most of the human population (Coulter *et al.*, 2008, 2010; Jenkinson *et al.*, 2010), and that Bandrowski’s base activates naive and memory T-cells (Gibson *et al.*, 2015). In this current study, T-cell clones were subsequently generated from 3 HLA-genotyped donors. Nitroso sulfamethoxazole, vancomycin, and Bandrowski’s base-responsive clones were detected from each donor and shown to proliferate in a dose-dependent manner. Carbamazepine, an anticonvulsant medication, associated with a variety of T-cell-mediated cutaneous hypersensitivity reactions as well as DILI (Ko *et al.*, 2011; Wu *et al.*, 2006) and the β-lactam antibiotic piperacillin, were the other training compounds. A statistically significant T-cell priming was observed in 1 donor against carbamazepine, whereas piperacillin-responsive T-cells were not detected. Notably, none of the donors in our experiments expressed HLA-A*31:01 and B*15:02 HLA alleles associated with carbamazepine hypersensitivity in different ethnic groups (Chen *et al.*, 2011; McCormack *et al.*, 2011). Carbamazepine was included in our set of training compounds not only because of the known HLA allele associations, but also because HLA-class II-restricted T-cells are detected in hypersensitive patients, including those expressing HLA-class I risk alleles. Thus, carbamazepine was deemed an important training compound to study, with a reasonable chance of detecting T-cell responses to the drug in non-HLA-typed blood donors. The absence of T-cell responses to piperacillin was somewhat surprising given that the drug forms adducts with proteins in exposed patients and that previous studies have shown the presence of β-lactam antibiotic-responsive T-cells in most healthy donors (Nhim *et al.*, 2013).

Of the 10 test molecules, none of the 6 test compounds with DILI liabilities stimulated T-cell responses in the dendritic cell-T-cell cultures; however, 2 of these 6 compounds (ciprofloxacin and erythromycin) were identified as potentially immunogenic compounds and therefore positive in our project through the generation of drug-specific T-cell clones. Ciprofloxacin is a broad-spectrum fluoroquinolone antibiotic effective against bacterial infections targeting the skin, bone, and respiratory tract. It is administered either orally or intravenously up to a maximum daily dose of 1500 mg in 2 divided doses for 5–7 days (Campoli-Richards *et al.*, 1988). Erythromycin is indicated for the treatment of a variety of infections in patients hypersensitive to penicillins and is in the top 10 ranking drugs associated with DILI (Teschke, 2018). It is administered either orally or intravenously up to a maximum daily dose of 4000 mg. Liver injury associated with both drugs is idiosyncratic, and may manifest as hepatocellular and cholestatic hepatitis (Andrade and Tulkens, 2011). For both drugs, the delayed onset of DILI following initial exposure, coupled with a rapid onset in a small number of reexposed patients, and presentation with symptoms of an allergic reaction (skin rash, eosinophilia) are indicative of an adaptive immune mechanism (Orman *et al.*, 2011). This work therefore demonstrates that T-cell activation assays have the potential to flag a concern for compounds, and this approach could be used during preclinical assessment to provide advanced warning of immunogenicity and/or triage compounds based on their potential for immunogenicity.

Although FDA warnings for hypersensitivity and/or DILI exist for iproniazid, nevirapine, nitrofurantoin and dacarbazine, our *in vitro* T-cell assay did not flag these compounds as immunogenic. Susceptibility toward nevirapine-induced skin and liver damage is associated with expression of a variety of HLA alleles (eg, B*35, B*58:01, C*04, and DRB1*01) (Cornejo-Castro *et al.*, 2015). Only 1 out of the 3 healthy donor samples utilized for the generation of drug-specific T-cell clones expressed HLA-B*35 and C*04:01 (Table 2). This highlights a limitation of this work that a large enough number of donors with differing HLA alleles would be best utilized to give maximal chance of identifying potential immunogenicity. Clinical data describing iproniazid, nitrofurantoin, and dacarbazine DILI are indicative of an immune pathogenesis, however no drug-responsive T-cells were detected.

The detection of immunogenicity for 2 compounds out of 6 compounds with DILI liabilities is a considerable proportion, especially given that there were no false positives for the 4 test drugs with low to no DILI liabilities (diphenhydramine, etoposide, mepivacaine, phenoxybenzamine); this may provide confidence to use this system to investigate preclinical compounds. Early identification of compounds with such a liability could have a significant impact, in terms of human safety both in healthy volunteer and patients, improving candidate quality and reducing drug attrition. This is particularly exemplified with ciprofloxacin, which would not have been flagged as a concern by typical hepatotoxicity-related screening assays (GSH adduct formation [Sakatis *et al.*, 2012], CYP3A4 metabolism-dependent inhibition [Sakatis *et al.*, 2012]; BSEP, or cytotoxicity assays [Schofield *et al.*, 2021]); thus, demonstrating potential added value of having such T-cell activation assays used for prospective assessments.

As well as demonstrating the potential for added value, there are still many limitations of these T-cell activation tools, with scope for future development. Firstly, whilst 2 drugs were identified by the generation of T-cell clones, this is very resource-intensive and time consuming, whereas no signal was identified for these drugs in the simpler and less resource-intensive dendritic cell-T-cell coculture proliferation assay. Another option will be to use these assays to investigate and understand events in the clinic (or findings in preclinical toxicology studies). In this circumstance, there would be knowledge of the drug-specific T-cell response that develops in patients with liver injury, in particular, the nature of the drug moiety that activates T-cells, the concentrations associated with the T-cell response and the pathway of T-cell activation, and any known HLA association identified or suspected in the clinic. With this information, it would be easier to model the T-cell response with PBMC from healthy donors to aid in the investigation of HLA-associations, thus providing information that may enable personalized use of drugs that carry this liability.

Moving forward, it is important to consider possible strategies to improve the sensitivity and confidence in these T-cell activation assays, to potentially be able to deploy these in prospective immunogenicity screening. One needs to consider the source of PBMC, and the number of donors needed to study to detect a drug-specific response. In this respect, it might be possible to utilize PBMC from multiple donors (eg, *n* = 100 or more), with different ethnic backgrounds covering all major HLA alleles associated with known immunological drug reactions, screened in parallel in multiwell culture plates, in the presence and absence of compound. However, this form of assay will suffer from the low precursor frequency of drug-responsive T-cells found in healthy donor blood. Alternatively, one could adopt different readouts to detect drug-specific responses (eg, ELIspot) or a multiomics approach to search for sensitive gene/protein changes in drug-treated PBMC from patients with immunological drug reactions, and then apply a panel of sensitive biomarkers of T-cell priming studies in healthy donors. One other important consideration is drug metabolism, particularly that which results in reactive metabolite formation, which is an established risk factor for forming a drug-derived antigen. The introduction of a metabolic component to such assays would enable the application of these assays to compounds that have been identified to undergo reactive metabolite formation during screening (such as those test compounds used in this work), to investigate the likelihood that this would activate effector T-cells, and could potentially inform on which HLA alleles would render individuals more susceptible to this risk. In this respect, we have recently developed a strategy for metabolite generation in immune cultures using human hepatocytes as metabolite generators (Ali *et al.*, 2023). However, new approach methodology is required to integrate a similar system to the assays described herein. Lastly, whilst the data generation on these compounds has been encouraging, in order to more fully understand the predictivity, utility, and confidence in such assays, a much larger set of compounds would need to be evaluated.

To conclude, this is the first study to use primary human immune cells to predict the intrinsic T-cell immunogenicity of blinded small molecule compounds associated with immunologically mediated liver injury in humans. This strategy detected T-cell responses to 5 compounds (3 training and 2 test compounds). Importantly, T-cell responses were not detected to the compounds with lower DILI liabilities. The currently available methods could potentially be applied to study compounds in late phase development to understand and investigate events that occur in clinic, or if second in line compounds with similar structures are being considered for human use. Further optimization of immune regulatory parameters and readouts for T-cell activation, together with the utilization of a large bank of PBMC from multiple donors, and potentially a metabolic component, could potentially enable use of such assays for prospective assessments.

## Supplementary Material

kfad101_Supplementary_Data

## Notes

The authors certify that all research involving human subjects was done under full compliance with all government policies and the Helsinki Declaration.
