# Supplementary material for: A blinded in vitro analysis of the intrinsic immunogenicity of hepatotoxic drugs: implications for preclinical risk assessment
Source: Toxicol Sci. 2023 Oct 3;197(1):38–52. doi: 10.1093/toxsci/kfad101 (PMC10734620; doi:10.1093/toxsci/kfad101)
Supplement: kfad101_Supplementary_Data [file kfad101_supplementary_data.pdf]

Supplementary Figure 1

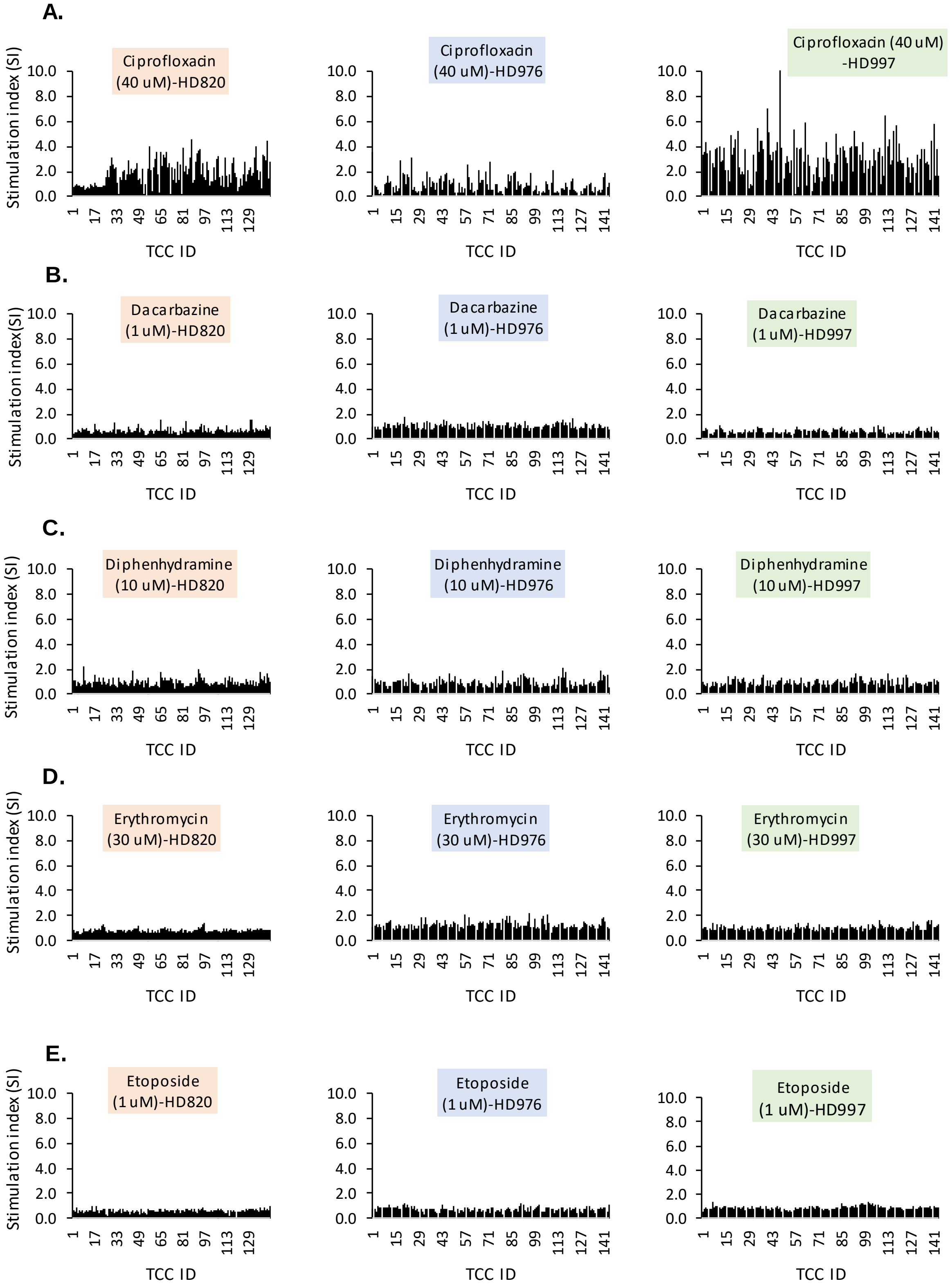

Supplementary figure 1 continued

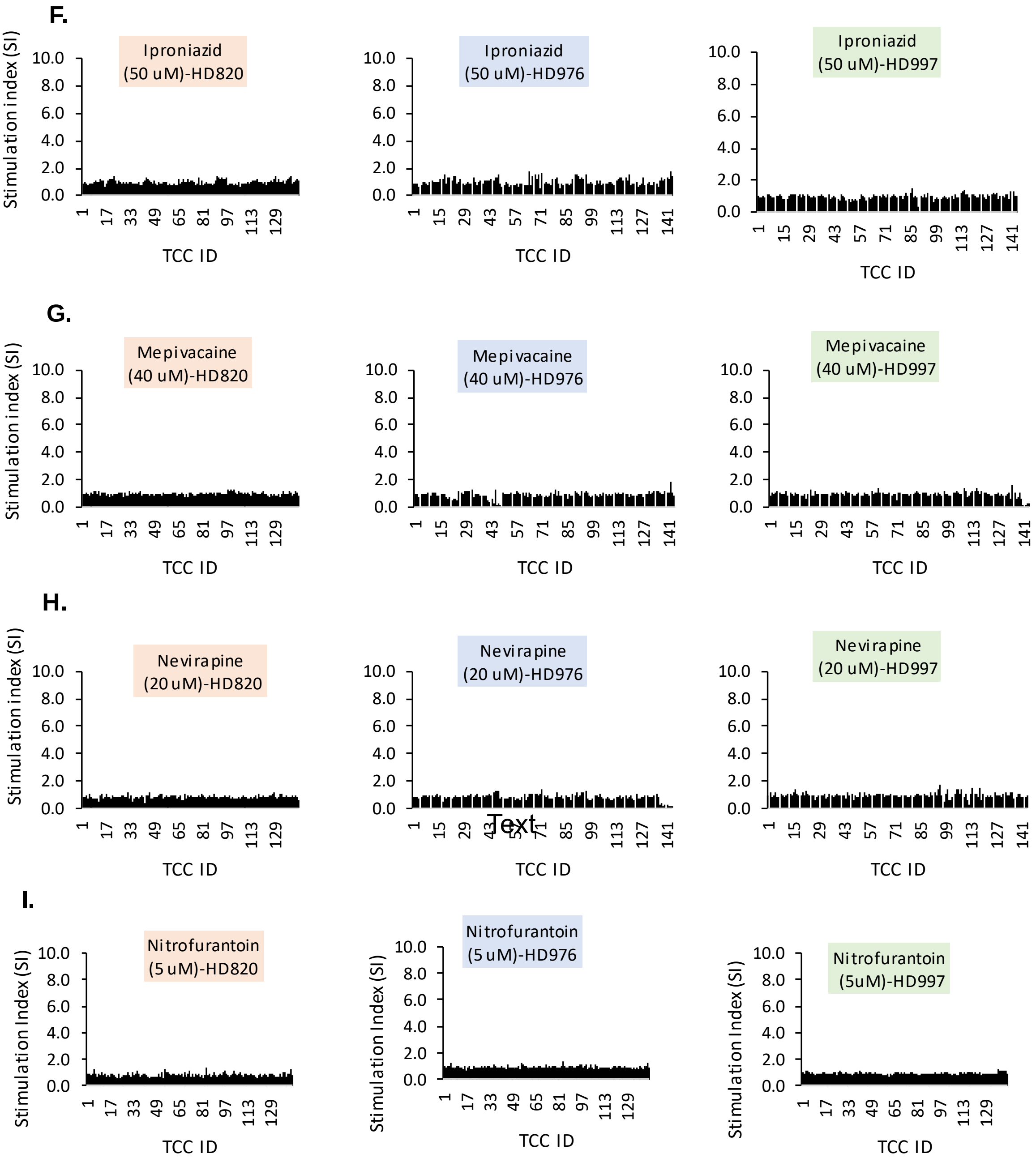

Supplementary Figure 1: Generation of T-cell clones from test compound-exposed PBMC (complete initial test results). PBMC from 3 healthy donors were cultured with test compounds and supplemented with IL-2 (50 U/ml) for 14 days. T-cell clones were generated from the T-cell lines by serial dilution and repetitive mitogen stimulation. T-cells (0.3 – 3 cells/well) were stimulated with irradiated allogenic PBMC (5 x 104/well) and phytohemagglutinin (1μg / ml). Well growing cultures were subjected to a second round of expansion then tested for drug-specific T-cell proliferation. Proliferation was measured by the addition of 3H-thymidine followed by scintillation counting. Clones displaying an SI of 2 or above were further expanded and subjected to dose-response studies.
